# Cytokine and Cancer Biomarkers Detection: The Dawn of Electrochemical Paper-Based Biosensor

**DOI:** 10.3390/s20071854

**Published:** 2020-03-27

**Authors:** Song Wei Loo, Tze-Sian Pui

**Affiliations:** School of Chemical and Biomedical Engineering, Nanyang Technological University, Singapore 639798, Singapore; songwei.loo@ntu.edu.sg

**Keywords:** electrochemical detection, paper-based device, biosensor, cytokine, cancer biomarkers, point-of-care device

## Abstract

Although the established ELISA-based sensing platforms have many benefits, the importance of cytokine and cancer biomarkers detection for point-of-care diagnostics has propelled the search for more specific, sensitive, simple, accessible, yet economical sensor. Paper-based biosensor holds promise for future in-situ applications and can provide rapid analysis and data without the need to conduct in a laboratory. Electrochemical detection plays a vital role in interpreting results obtained from qualitative assessment to quantitative determination. In this review, various factors affecting the design of an electrochemical paper-based biosensor are highlighted and discussed in depth. Different detection methods, along with the latest development in utilizing them in cytokine and cancer biomarkers detection, are reviewed. Lastly, the fabrication of portable electrochemical paper-based biosensor is ideal in deliberating positive societal implications in developing countries with limited resources and accessibility to healthcare services.

## 1. Introduction

Cytokines are microproteins secreted by cells that play a crucial role in cell signaling as immunomodulating agents, typically in the activation of immune response against pathogens [1,2]. They can be categorized into few functional classes, being pro-inflammatory, anti-inflammatory, or adaptive immune response for instance. Since cytokines exist as non-structural proteins, their induced biological properties were, and still are the gold standards for defining them [3]. The classification of cytokines had been compiled in Table 1 based on their triggered immune response, along with the specific roles of individual cytokines that depend on cell type and location as shown in Table 2. Although cytokines can be produced by numerous cell populations, the main producers are macrophages and helper T cells [1,2,4]. Many individual cytokines can exhibit their hallmark pleiotropic and overlapping activities by activating multiple signaling pathways, thus contributing to different functions [4,5]. In contrast, different cytokines may also demonstrate redundancy wherein they share identical receptor chains and have similar functions [1,4,5].

Germane to their involvement in immune system, cytokines are potential biomarkers that can be utilized in the monitoring of disease activity and their subsequent severity [6,7,8]. Owing to the influential role that cytokines play in cell-mediated immunity, it can be considered as potential therapeutic targets for the treatment of various infectious, inflammatory, neurological, and even neoplastic diseases [9,10,11]. A dysregulation in cytokine levels has been correlated with the onset of several types of cancer such as gastric cancer and colorectal cancer, thus its role exhibits promising quality as cancer biomarker to support diagnosis [12,13]. Apart from this, cytokines can also serve as prospective targets for the development of vaccines [14]. Hence, their detection and quantification can provide diagnostic value of great importance. 

At the time of writing, the broad definition of cytokine includes interferons, adipokines, interleukins, and tumor necrosis factors [3,4]. Being the only member of the type II class of interferons, interferon-gamma (IFN-γ) often appears to be the conundrum [3]. Although this particular cytokine is vital for our immune defense against microorganisms such as *Mycobacterium tuberculosis*, it is also one of the causative factors in the pathogenesis of various autoimmune diseases [3]. Therefore, the sensitive detection of human IFN-γ is important for the precise diagnosis of diseases by clinicians. Currently, the sensitivity of the detection of IFN-γ at serum level using an enzyme-linked immunosorbent assay (ELISA) kit is reported to be 15 pg/mL and the standard curve range was 15-2000 pg/mL [15].

Other than the interferons, adipokines are low molecular weight, pleiotropic cytokines that are commonly associated with obesity [16]. Leptin, the first member of the adipokine family, is found to act as a body weight central regulator by linking neuroendocrine function with nutritional status [16,17]. It was reported that obese individuals have elevated circulating leptin level, yet fails to reduce food intake and increase energy expenditure due to resistance in the hypothalamus, resulting in body weight gain [17]. Few other members of the adipokine family, namely adiponectin, progranulin (PGRN), and lipocalin-2 (LCN2) are also found to exhibit vital links between immune system and obesity, thus making adipokines attractive biomarkers for obesity-related diseases, such as Type 2 diabetes mellitus, osteoarthritis, and rheumatoid arthritis [18,19,20].

Apart from that, interleukin 6 (IL-6) is a pro-inflammatory cytokine and its overexpression is relevant to numerous types of cancers, such as gastrointestinal cancer [21], head and neck carcinomas [22], and colorectal cancer [23]. It was reported that IL-6 is present at a very low concentration of approximately 6 pg mL^−1^ in healthy individuals [24,25], therefore making its detection and early diagnosis of diseases to be an extremely challenging task. Few methods have been reported for fabricating IL-6 biosensors with remarkable sensitivity and selectivity [26,27,28]. The sensitivity of the detection of IL-6 at serum level using ELISA kit is reported to be 4 pg/mL and the standard curve range was 4-500 pg/mL [15]. 

Furthermore, tumor necrosis factor alpha (TNF-α) is a pro-inflammatory cytokine and its dysregulation has been linked to the onset of various human diseases, including cardiovascular diseases [29], rheumatoid arthritis [30], Alzheimer’s disease [31], and psoriasis [32]. Hence, it is crucial to measure TNF-α for the in-depth understanding of inflammation, thus its role has been extended to biomarkers for the diagnosis and monitoring of disease severity [33,34].

Although cytokines have been considered as cancer biomarkers hitherto, few other glycoproteins, typically carcinoembryonic antigen (CEA) and tissue inhibitor of metalloproteinase 1 (TIMP1), are involved in cellular adhesion that is commonly produced in gastrointestinal tissue and their abnormal levels are reported in serum of lung, colorectal, and breast cancer [35,36]. Therefore the detection of CEA or TIMP1 as cancer biomarkers is crucial for cancer diagnosis, or more specifically, for the monitoring of patients who suffer from cancer which produce CEA after and before getting chemotherapy, surgery, radiation, or combination of all [36,37]. 

In this review, we shall provide detailed insight into the various factors that can influence the design of an electrochemical paper-based biosensor, along with the latest development in utilizing them in cytokine and cancer biomarkers detection. The selection criteria for this type of biosensors are listed as follow: (i) fabrication steps that involve the use of any paper-based substrate, (ii) fabricated sensors that utilize electrochemical methods as their mode of detection, and (iii) fabricated sensors that detect only cytokines or cancer biomarkers. Therefore, any biosensors that do not fulfill all three of the criteria shall not be reviewed. 

## 2. Methodology for Cytokine and Cancer Biomarkers Quantification

Traditionally, cytokine can be measured and quantified using several methods, namely ELISA, radioimmunoassay (RIA), biochemical assays, and multiplex array [38,39]. Among the list the conventional ELISA has proven to be the benchmark in most biophysical methods [38]. It was introduced in the 1970s to replace the use of radioactive isotopes in RIA described by Yalow and Berson in 1959—a discovery that won Yalow the 1977 Nobel Prize in Physiology or Medicine [38,40]. ELISA offers high specificity and sensitivity for cytokine detection in biological samples such as serum or cell supernatant [38,39]. Furthermore, the results obtained from ELISA have high reproducibility and generally accurate. However, the drawback in using ELISA is that it can only measure a single type of cytokine present in each sample for each trial as compared to other sensing platforms. Although the cost of ELISA is still affordable, a lower cost sensing platform would naturally be more beneficial.

In recent years, multiplex arrays have been developed from traditional ELISA in order to address the limitations in ELISA with the aim of performing multiple cytokines and cancer biomarkers measurement in the same given serum sample at a time [39]. These innovative arrays are available in varying formats established from the fundamental understandings and utilization of flow cytometry and chemiluminescence technology [38,39]. Although having lower sensitivity, these multiplex arrays have countless advantages over singleplex ELISA, including minimal sample volume requirement, broader quantification range for each cytokine, and vast reduction in both assay time and cost [41]. Although the established ELISA-based sensing platforms have many benefits, an alternative sensing platform with lower cost would naturally be more favorable.

Various unconventional modern biosensors developed for the detection of cytokines were reported in recent years in the forms of capacitance-based sensor, waveguide grating optical sensor, and field-effect transistor (FET) sensor [42,43,44,45]. For capacitance-based sensor, the detection of cytokines is based on the relative change in their capacitive values, whereas optical biosensors are based on the changes in refractive index that resulted from bound analytes [43,44]. Research on FET sensors have shed new light on the development of novel biosensor as they have been employed to study on cellular bioelectricity by performing electrophysiology measurements [42,45]. 

It has been highlighted that there is a pressing need for the development of a specific, sensitive, simple, accessible, yet economical diagnostic method. In this case, the development of paper-based biosensors have caught the attention of researchers owing to their idiosyncratic properties, accompanied by the portability of the device which can in turn, provide rapid analysis and data without the need to conduct in a laboratory [46]. 

## 3. Strategies for Designing Electrochemical Paper-Based Biosensors 

The approach to design an electrochemical paper-based biosensor that serves as a paragon of sensitivity, specificity, simplicity, and affordability can be challenging, yet rewarding and worthwhile. There are few aspects that need to be considered prior to fabricating an electrochemical paper-based biosensor, and the aspects were clearly stated and elaborated with detailed explanations in the following context.

### 3.1. Selection of Paper Material

The use of paper in fabricating a biosensor has shown potential owing to its ubiquity and unique properties, for instance, its porosity, surface affinity towards varying analytes, and the wicking rate of liquid [47]. As a matter of fact, paper provides a thin layer of water that can deliver analytes to electrode surface, thus enhancing the surface area and conductivity of the fabricated biosensor [48,49]. Evidently, different paper materials as substrates have different properties in terms of pore size, thickness, grades, and flow rate. As a result, the selection of paper material is crucial so that the properties of the selected paper substrate can be fully utilized in order to maximize detection sensitivity [50]. There is a myriad of paper substrates ranging from filter paper to varying grades of office paper that can be chosen from and utilized depending on the specific application required; each variety, in turn, offers different characteristics [51]. Ideally, paper substrate made up of thinner material is more favorable for several fabrication methods such as inkjet-, Stencil-, and wax-printing as it requires smaller amount of ink or wax to be deposited onto the surface of the substrate for penetration through the nitrocellulose membrane of paper in order to create a hydrophobic zone in the biosensor, which in turn, reduces production cost [50]. The most widely used paper substrate to date is the Whatman grade 1 chromatographic filter paper. It gains popularity from its structural uniformity, high alpha-cellulose content (> 98%) and smooth surface, which provides quality assurance and guarantees data reproducibility and uniformity [49]. Apart from that, various studies revealed that the Whatman grade 1 chromatographic filter paper is suitable for the immobilization of DNA, proteins, and enzymes owing to its extraordinary non-specific binding affinity towards bioactive molecules [52,53,54,55,56].

### 3.2. Design of Two- and Three-Dimensional Biosensors

**Figure 1:**
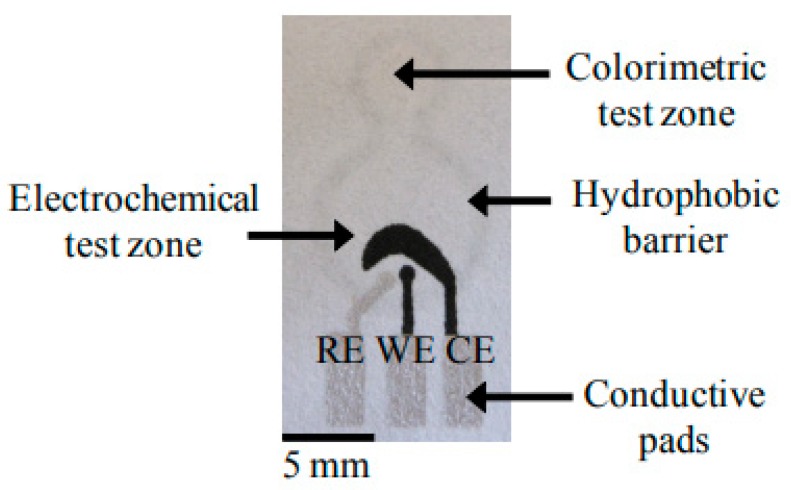
A 2D paper-based microfluidics fabricated using photolithographic method [61]. Total iron was analyzed by colorimetric method involving the formation of red-colored complex between iron(II) and 1,10-phenanthroline as shown in the center of the figure. Abbreviations: RE, reference electrode; WE, working electrode; CE, counter electrode.

**Figure 2:**
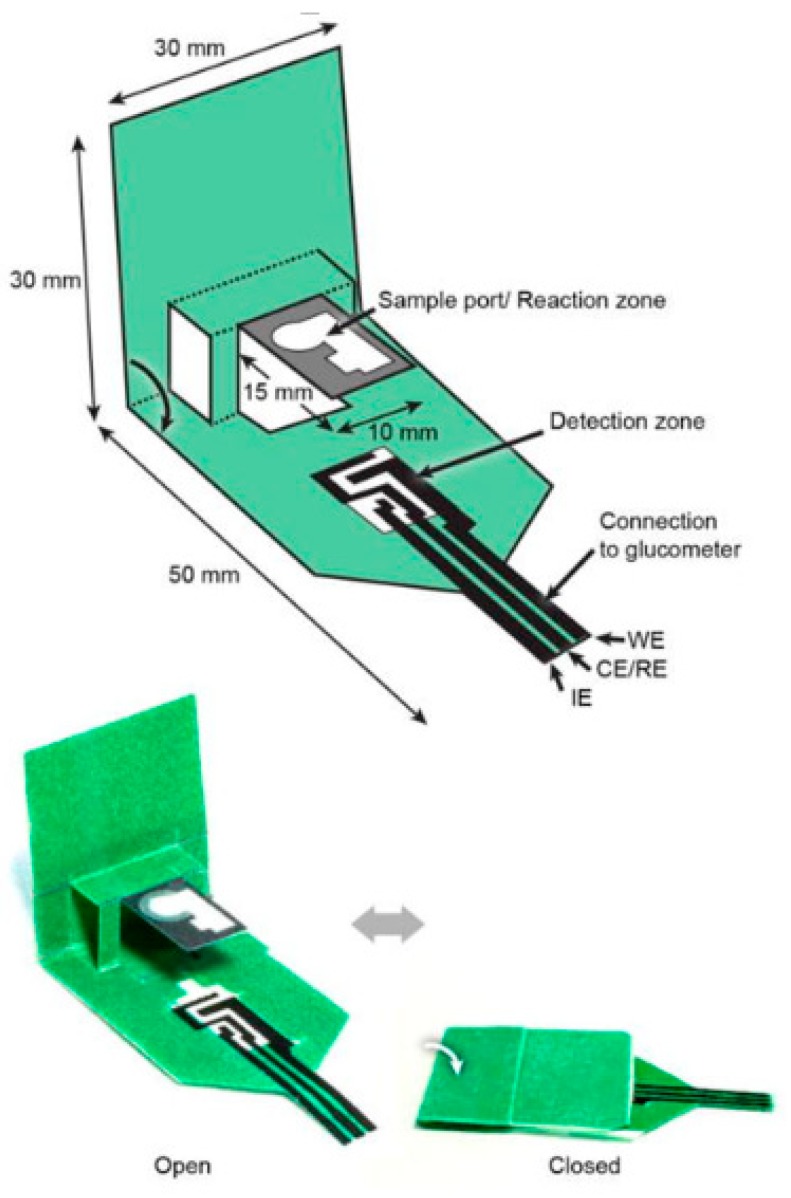
A 3D “pop-up” electrochemical paper-based sensor developed for analysis of beta-hydroxy-butyrate [62].

Back in 2007, Prof. Whitesides and his research team introduced the two-dimensional (2D) concept in the fabrication of paper-based sensors, for which multiple analytes can be simultaneously detected using a single sample pool [57]. A more sophisticated yet simple three-dimensional (3D) paper-based sensor was developed in 2016 to promote more complex and complicated operations [58]. The main noticeable dissimilarity between 2D and 3D paper-based sensors is none other than the way the electrodes are being displayed. For a 2D sensor, a three electrodes system is being implemented and fabricated onto a piece of paper substrate. In contrast, a paper is folded to form a specific configuration somewhat like origami, whereby the working electrode (WE) is being fabricated onto one segment of the paper while both the reference electrode (RE) and counter electrode (CE) are constructed on another in a 3D sensor [59]. By comparing both 2D sensor and 3D sensor, the latter has the edge over the former as fluid can travel freely in both vertical and horizontal directions in a 3D sensor, thus demonstrating a highly homogeneous coloration covering the whole surface area of the paper reaction zones [60]. Examples of a 2D sensor and a 3D sensor are illustrated in Figure 1 and Figure 2, respectively.

### 3.3. Formation of Hydrophobic Walls

**Figure 3:**
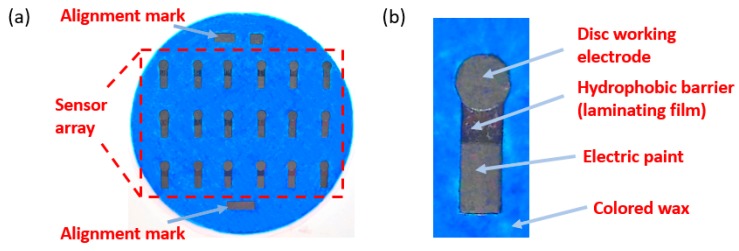
Pictures of a wax-printed electrochemical paper-based sensor: (**a**) Optical image of working electrode array on a 90 mm diameter filter paper; (**b**) disc working electrode (4 mm diameter) which is surrounded by colored wax and a hydrophobic barrier on top [67].

To form a microfluidic channel, the path of the flowing solutions and the hydrophilic zones can be well defined by forming hydrophobic walls utilizing the porous matrix within the cellulose membrane of a paper substrate [49]. This form of hydrophobic patterning can effectively prevent the fluid solution from overflowing or backflowing from the paper sensor [63]. There are some common fabrication techniques that can be applied to form hydrophobic walls on paper substrate, for example, inkjet-, wax-, screen-, laser-printing, along with more costly photolithography [64]. Although each technique has its own pros and cons, wax printing is much more desirable and stands out from other techniques. This technique is quite straightforward and capable of forming both hydrophobic and hydrophilic zones on cellulose matrix [65]. The fabrication steps of wax printing involve printing the wax onto the paper substrate, heating the wax till it melts, then spreading it precisely and uniformly to form hydrophobic walls across the surface of the paper substrate [66]. A wax-printed electrochemical paper-based sensor is depicted in Figure 3.

### 3.4. Surface Modification of Electrodes

Owing to the extensive matrix of nitrocellulose membrane of a paper, it serves as the foundation for the deposition of the electrodes’ conductive and redox materials in a paper-based biosensor [49]. In fact, the sensitivity and conductivity of the electrode, along with the cost of the fabricated biosensor are highly dependent on the selection of specific electrode material. Consequently, the materials selected for the fabrication of WE, RE, and CE may differ from each other depending on the target cytokine to be analyzed and the type of analysis required [68]. These electrodes can be fabricated using conducting pastes comprised of graphene, gold, silver, carbon and heavy metal. Among all materials, carbon paste has been widely used in the fabrication of CE and WE due to its lower interference in the screen-printing technique compared to other materials. As for RE, silver paste is more favorable owing to its consistent potential and stability for electrochemical detection, which enhances the signal output [69]. 

In order to maximize the sensitivity, stability, and reproducibility of cytokine detection, the surface of the fabricated electrodes can be subjected to further modifications to promote higher binding capacity and more rapid recognition of target cytokine as compared to planar, stationary surfaces [58]. One of the simpler surface modification pathways involves the use of nanoparticles (NPs). The utilization of noble metal nanoparticles (NMNPs) can further expand the electrode’s surface and improve the surface conductivity by acting as charge carriers and catalysts for electrochemical analysis, thus amplifying the resulting signal detected [69]. Moreover, the incorporation of NMNPs such as gold nanoparticles (AuNPs) are reported to enhance biocompatibility as well [70]. Such surface modification can be characterized using several common techniques, such as electrochemical impedance spectroscopy (EIS) for the determination of an electrochemical system’s response to an applied potential, scanning electron microscopy (SEM) for surface composition, and transmission electron microscopy (TEM) for internal composition.

Apart from NMNPs, non-NMNPs such as carbon nanotubes and graphene have also been reported as alternatives for surface modification of electrodes for improved detection [71,72,73,74,75]. For instance, an amplified signal was recorded when graphene oxide nanoflakes and zeolite nanocrystals were introduced onto the electrode surface of a paper sensor due to high electron transfer kinetics and larger surface area of the nanocomposites [76]. In another work that focused on the detection of bisphenol A (BPA), a type of surface modification involving the incorporation of gold nanoparticle layer with multi-walled carbon nanotubes (MWCNTs) was reported and showed promising enhancement effects to the oxidation of BPA [77].

### 3.5. Antibody-Based Versus Aptamer-Based Approach

There are two approaches for the design of electrochemical paper-based biosensor specifically for the detection of cytokine and cancer biomarkers: antibody-based or aptamer-based. 

**Figure 4:**
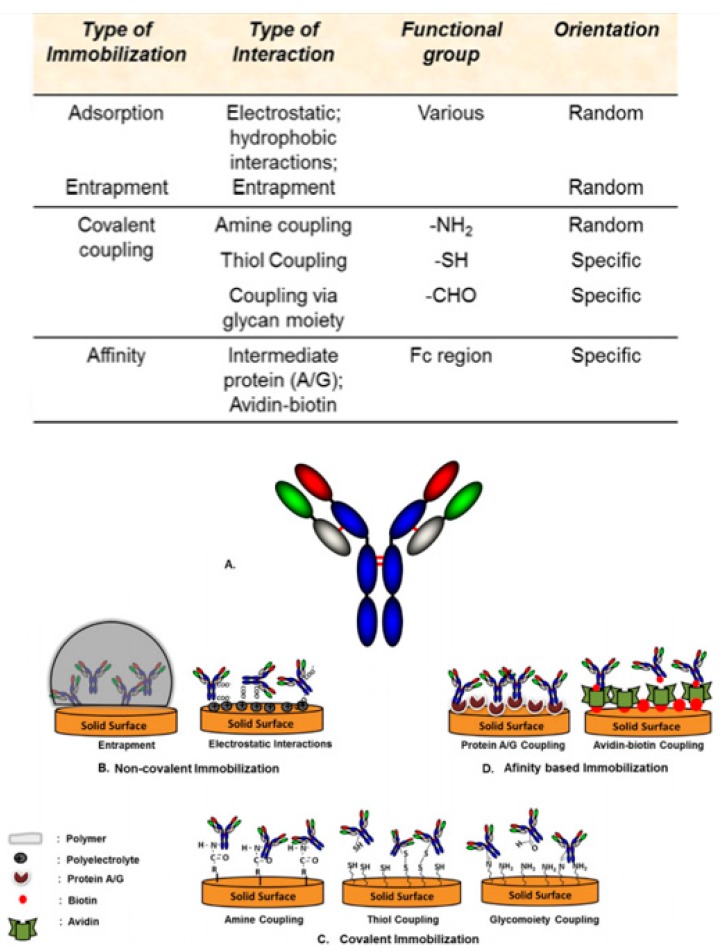
Overview of different functional groups utilized for different modes of antibody immobilization on to the biosensor’s surface and schematic representation of different immobilization techniques used to immobilize antibodies on to a biosensor’s surface. (**a**) Typical antibody structure; (**b**) non-covalent immobilization of antibodies on to a biosensor’s surface through entrapment and electrostatic interactions; (**c**) covalent immobilization of antibodies on to a biosensor’s surface through various functional groups such as aldehyde, thiol, and amine groups; (**d**) affinity-based immobilization of antibodies on to a biosensor’s surface via protein A/G coupling and avidin-biotin coupling [78].

Since the groundbreaking discovery of the chemical structure of antibodies back in 1959, they have been extensively studied and used as diagnostic and therapeutic tools owing to their exquisite specificity for their cognate antigen [78]. The performance of a fabricated antibody-based biosensor is dependent on three factors: (i) the accessibility of the bioactive molecules to the relevant analyte in sample; (ii) its ability to immobilize bioactive molecules; and (iii) degree of non-specific adsorption to the solid support. In order to limit non-specific adsorption and to optimize immobilization of antibodies, the physicochemical properties of the biosensor’s surface play a key role in determining the sensitivity, detection limit and overall performance of the biosensor [79,80]. Apparently, the asymmetric macromolecular antibodies can adsorb to the surface of the biosensor in various orientations. To optimize sensor performance, immobilization should be made available through the constant fragment (Fc) region so that the binding site of the antibody can be made available for maximum interaction with the cognate antigen [81]. Minimal structural modification and favorable orientation of antibodies upon immobilization on the biosensor’s surface can therefore further optimize the performance of a biosensor with improvement performance factors reported to be as high as 200-fold compared to random immobilization [80]. For example, a more stable immobilization resulting in specific orientation can be achieved when the thiol group is utilized for surface immobilization of antibody [80]. Albeit having specific orientation, this method is restricted; it produces monovalent antibodies, and harsh reduction conditions might inactivate antibody fragments due to unfavorable and unintentional reduction of internal disulfide bonds since antibody chains are linked through disulfide bridges [80]. An overview of different functional groups utilized for different modes of antibody immobilization on to the biosensor’s surface, as well as the schematic representation of different immobilization techniques used to immobilize antibodies is shown in Figure 4. 

Although antibody-based sensors have remarkable sensitivity, these biosensors require the use of expensive antibodies, where large amount of purified antigen is required to inject into animals to produce antibodies [82]. Therefore, the need to develop novel methods for improved performance cannot be overlooked. Lately, much attention has been shifted onto the development of aptamer-based sensors as new biosensing devices [2,82]. An aptamer is a single-stranded nucleic acid that is capable of binding selectively to a target biomolecule based on its well-defined tertiary structures. Compared to antibodies, aptamers are more stable, thus making them an ideal candidate to be incorporated into biological diagnostic devices [83,84]. Therefore, an aptamer-based biosensor is much more favorable compared to an antibody-based biosensor.

## 4. Electrochemical Detection of Paper-Based Biosensors

In this segment we shall move on to inspect a few examples on different electrochemical-based detection techniques of paper-based biosensor posterior to our fundamental understandings on the factors affecting the design of this type of sensor. Commonly used electrochemical methods for the fabrication of paper-based biosensors include the various forms of voltammetry (differential pulse, cyclic, linear sweep, stripping), impedance spectroscopy, and electrochemiluminescence detection. We shall focus our review encircling their applications in cytokine and cancer biomarkers detection.

### 4.1. Voltammetry 

**Figure 5:**
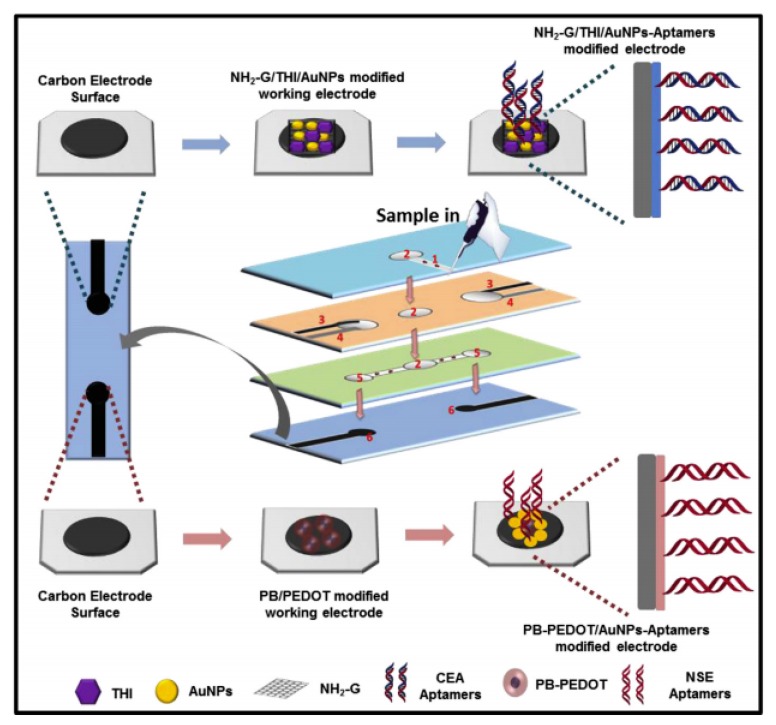
Fabrication and modification process of the multiplex electrochemical paper-based aptasensor [85].

Recently, a study reported the fabrication of a label-free microfluidic paper-based aptasensor for sensitive and simultaneous detection of cancer biomarkers CEA and neuron-specific enolase (NSE) as depicted in Figure 5. The paper-based biosensor was fabricated through wax- and screen-printing. The working electrodes were modified with amino functional graphene (NG)-Thionin (THI)-gold nanoparticles (AuNPs) (NG-THI-AuNPs) and Prussian blue (PB)-poly(3,4-ethylenedioxythiophene) (PEDOT)-AuNPs (PB-PEDOT-AuNPs) nanocomposites, respectively [85]. Experimental results obtained from cyclic voltammetry (CV) and differential pulse voltammetry (DPV) revealed that the aptasensor demonstrated remarkable linearity in ranges of 0.01-500 ng mL^−1^ for CEA and 0.05-500 ng mL^−1^ for NSE, respectively, whereas the detection limit was determined to be 2 pg mL^−1^ for CEA and 10 pg mL^−1^ for NSE. Therefore, it was shown that paper-based biosensors offer an alternate platform for early cancer diagnostics, especially in resource-limited countries [85].

### 4.2. Impedance Spectroscopy

**Figure 6:**
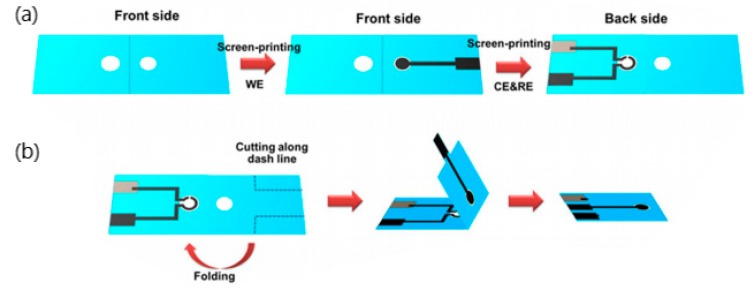
(**a**) Fabrication procedure of label-free paper-based impedance immunosensor for human interferon-gamma (IFN-γ), (**b**) 3D folding sequence [86].

The latest breakthrough was achieved in the form of an electrochemical label-free paper-based impedance biosensor. A graphene screen-printed paper electrode modified with polyaniline showed increased surface area for antibody immobilization and exceptional electrochemical conductivity, thus contributing to its enhanced sensitivity. A schematic illustration of the fabrication of this biosensor is shown in Figure 6. A linear relationship between logarithmic concentrations of human IFN-γ and impedance was determined in the range of 5-1000 pg mL^−1^ with detection limit of 3.4 pg mL^−1^. Surprisingly, the polyaniline-graphene modified electrodes displayed more than 30 times higher sensitive compared to common polyaniline-modified electrodes [86]. Furthermore, this system is low cost, requires minute sample volume and able to provide rapid analysis compared to traditional methods. It has excellent prospect to be developed as an alternative platform for human IFN-γ screening [86].

**Figure 7:**
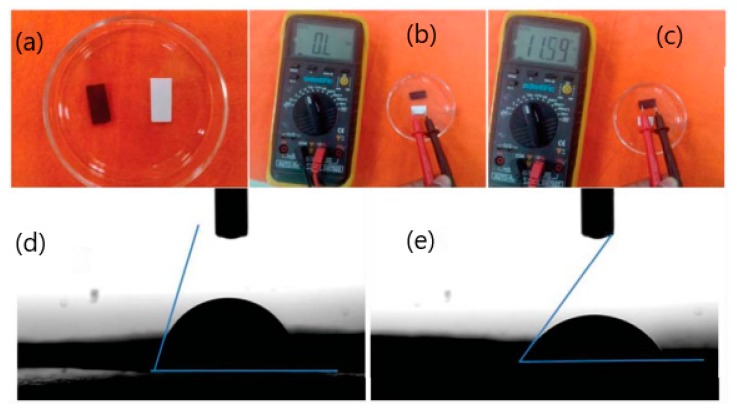
Photos of paper (white) and conducting paper strips (black) (**a**,**b**,**c**); contact angle measurement of conducting paper strips (**d**); (3-aminopropyl) triethoxysilane (APTES) functionalized conducting paper strips (**e**) [36].

Another study reported results relating to the fabrication of a paper-based impedimetric biosensor using a poly(3,4-ethylenedioxythiophene):poly(4-styrene sulfonate) (PEDOT:PSS) modified Whatman filter paper as depicted in Figure 7. This portable paper platform has been used for selective quantitative detection of the cancer biomarker CEA [36]. The results indicated that the fabricated paper-based biosensor has promising applications in cancer biomarker detection, having sensitivity of 3.6 Ω mL ng^−1^ with a lower detection limit of 2.68 ng mL^−1^ in the range of 6-20 ng mL^−1^ CEA estimation [36]. Currently, the sensitivity of CEA at the recommended 5 µg/L threshold is well documented [87,88]. However, a proposed cut-off of 2.2 ng/mL may provide an ideal balance of sensitivity and specificity [88]. 

### 4.3. Electrochemiluminescence Detection 

Electrochemiluminescence (ECL) detection is another sensitive analytical technique that combines electrochemical and luminescent methods. It is a process in which the emission of light is initiated by a redox reaction occurring at an electrode surface [89]. This detection technique has attracted much interest due to its distinctive advantages, for instance, high sensitivity, low background noise, and spatial controllability.

**Figure 8:**
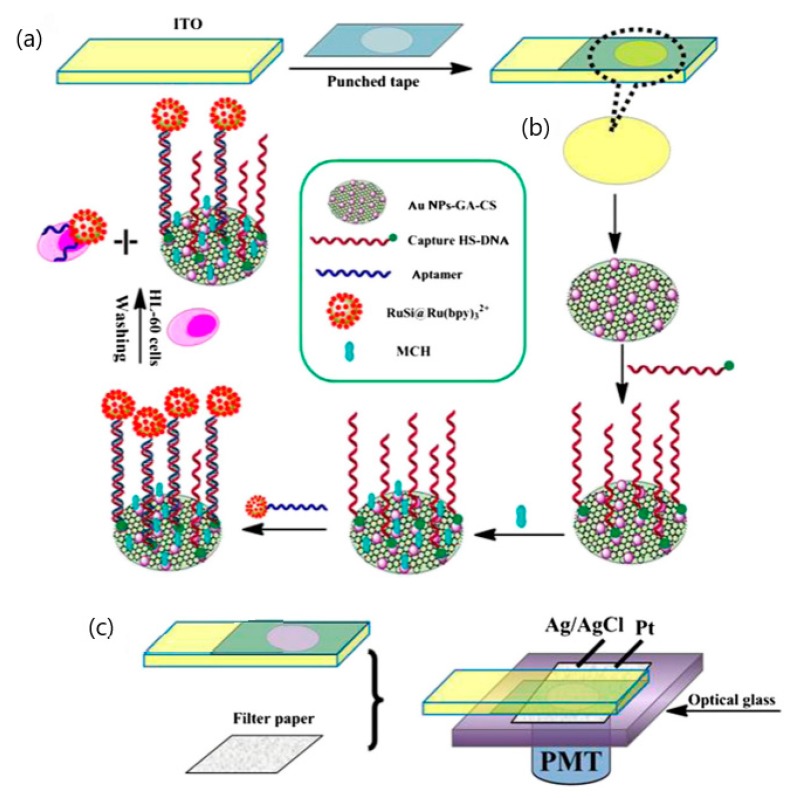
Schematic illustration of paper-based biosensor for Electrochemiluminescence (ECL) detection of human leukemia (HL-60) cells. (a) Fabrication of indium-tin oxide (ITO) electrode, (b) Sensing principle to HL-60 cancer cells based on [Ru(bpy)_3_]^2+^-conjugated silica nanoparticles as emitter on the surface of ITO electrode, (c) A platinum wire and an Ag/AgCl wire were attached on to the filter paper with a clasp and used as the counter electrode and reference electrode, respectively [90].

Xu and co-workers had reported the fabrication of a disposable paper-based ECL biosensor for the detection of human leukemia (HL-60) cells as shown in Figure 8. This ECL biosensor is developed using an aptamer-based approach and utilized porous filter paper as an electrochemical cell connected to a sheet of indium-tin oxide WE modified with gold nanoparticles and graphene [90]. The aptamer of HL-60 cancer cells is tagged with [Ru(bpy)_3_]^2+^-conjugated silica nanoparticles. This detection limit for this fabricated disposable testing platform could go down to 56 cells per milliliter [90]. A compilation of various electrochemical-based detection techniques used and their respective criteria along with the results obtained was tabulated and illustrated in Table 3.

## 5. Pros and Cons of Electrochemical Paper-Based Biosensors

Apart from the various advantages of using electrochemical paper-based biosensors, there are some drawbacks that need to be dealt with appropriate measures. The advantages and disadvantages of paper-based biosensors over ELISA-based sensing platforms were being tabulated and presented in Table 4.

The handling of paper-based biosensors is easy and does not require highly trained personnel. A study reported that a paper-based biosensor using bioactivated multi-walled carbon nanotubes is about 20 times cheaper and over 10 times faster than ELISA, along with its maximum detection limit which is approximately 50 times higher than ELISA [91]. The main challenge associated with the application of paper-based biosensors is to preserve the activity of biomolecules stored in the pores of the paper device, especially for enzymes and antibodies which may be prone to oxidation by air when moist [92]. Therefore, it is advisable to store the paper-based biosensors in dry, sealed containers or polybags to prevent or slow down the degradation of biomolecules.

## 6. Limitations and Future Perspective

The main challenge for the fabrication of this type of biosensors is its reliability and ability to detect multiple cytokines or other biomarkers simultaneously in complex biological samples. Thus, future studies should focus on addressing the above issues and increasing the current shelf-life of these biosensors in order to promote future commercialization for the sensitive detection of cytokines and cancer biomarkers.

Moreover, paper-based biosensors can also be utilized in cytokine profiling among individuals in order to study the development of cytokine expression in humans. Unlike most biomarkers, cytokines are directly involved in mediating inflammation, and their measurement may predict the likelihood of immune response, which in turn, is crucial against pathogen infections.

## 7. Summary

Cytokines are potential biomarkers that can be detected and measured owing to their influential role in cell-mediated immunity. Although various traditional and unconventional methods have been developed for cytokine and cancer biomarkers detection and measurement over the years, the development of electrochemical paper-based biosensors have caught the attention of researchers owing to their idiosyncratic properties, device portability, specificity, sensitivity, simplicity, accessibility, yet being economical. Additionally, it provides rapid analysis and data without the need to enter a laboratory. Therefore, the development of electrochemical paper-based biosensor is important, especially in deliberating positive societal implications in developing countries with limited resources and accessibility to healthcare services. Apart from this, this type of biosensors is not restricted to the analysis of cytokines and cancer biomarkers; their applications have been incorporated into many fields, such as environmental and food testing. 

## Figures and Tables

**Table 1:** Classification of cytokines based on their triggered immune response [4].

Immune Response	Family	Members
Adaptive immunity	Common γ chain receptor ligands	IL-2, IL-4, IL-7, IL-9, IL-15, IL-21
	Common β chain (CD131) receptor ligands	IL-3, IL-5, GM-CSF
	Shared IL-2β chain (CD122)	IL-2, IL-15
	Shared receptors	IL-13 (IL-13R–IL-4R complex) TSLP (TSLPR–IL-7R complex)
Pro-inflammatory	IL-1	IL-1α, IL-1β, IL-1ra, IL-18, IL-33, IL-36α, IL-36β, IL-36γ, IL-36Ra, IL-37 and IL-1Hy2
	IL-6	IL-6, IL-11, IL-31, CNTF, CT-1, LIF, OPN, OSM
	TNFα	TNFα, TNFβ, BAFF, APRIL
	IL-17	IL-17A-F, IL-25 (IL-17E)
	Type I IFN	IFNα, IFNβ, IFNω, IFNκ, Limitin
	Type II IFN	IFNγ
	Type III IFN	IFNλ1 (IL-29), IFNλ2 (IL-28A), IFNλ3 (IL-28B)
Anti-inflammatory	IL-12	IL-12, IL-23, IL-27, IL-35
	IL-10	IL-10, IL-19, IL-20, IL-22, IL-24, IL-26, IL-28, IL-29

Abbreviations: CNTF, ciliary neurotrophic factor; CT-1, cardiotrophin-1; GM-CSF, granulocyte macrophage-colony stimulating factor; IFN, interferon; LIF, leukemia inhibitory factor; OPN, osteopontin; OSM, oncostatin M; TNFα, tumor necrosis factor α; TSLP, thymic stromal lymphopoietin.

**Table 2:** Specific roles of individual cytokines that depend on cell type and location [4].

Class of Cytokines	Specific Cytokines	Main sources	Target CELL	Major Function
Interleukins	IL-1	Macrophages, B cells, DCs	B cells, NK cells, T cells	Pyrogenic, pro-inflammatory, proliferation and differentiation, BM cell proliferation
	IL-2	T cells	Activated T and B cells, NK cells	Proliferation and activation
	IL-3	T cells, NK cells	Stem cells	Hematopoietic precursor proliferation and differentiation
	IL-4	Th cells	B cells, T cells, macrophages	Proliferation of B and cytotoxic T cells, enhances MHC class II expression, stimulates IgG and IgE production
	IL-5	Th cells	Eosinophils, B cells	Proliferation and maturation, stimulates IgA and IgM production
	IL-6	Th cells, macrophages, fibroblasts	Activated B cells, plasma cells	Differentiation into plasma cells, IgG production
	IL-7	BM stromal cells, epithelial cells	Stem cells	B and T cell growth factor
	IL-8	Macrophages	Neutrophils	Chemotaxis, pro-inflammatory
	IL-9	T cell	T cell	Growth and proliferation
	IL-10	T cell	B cells, macrophages	Inhibits cytokine production and mononuclear cell function, anti-inflammatory
	IL-11	BM stromal cells	B cells	Differentiation, induces acute phase proteins
	IL-12	T cells	NK cells	Activates NK cells
				
Tumor necrosis factors	TNF-α	Macrophages	Macrophages	Phagocyte cell activation, endotoxic shock
		Monocytes	Tumor cells	Tumor cytotoxicity, cachexia
	TNF-β	T cells	Phagocytes, tumor cells	Chemotactic, phagocytosis, oncostatic, induces other cytokines
Interferons	IFN-α	Leukocytes	Various	Anti-viral
	IFN-β	Fibroblasts	Various	Anti-viral, anti-proliferative
	IFN-γ	T cells	Various	Anti-viral, macrophage activation, increases neutrophil and monocyte function, MHC-I and -II expression on cells
Colony stimulating factors	G-CSF	Fibroblasts, endothelium	Stem cells in BM	Granulocyte production
	GM-CSF	T cells, macrophages, fibroblasts	Stem cells	Granulocyte, monocyte, eosinophil production
	M-CSF	Fibroblast, endothelium	Stem cells	Monocyte production and activation
	Erythropoietin	Endothelium	Stem cells	Red blood cell production
Others	TGF-β	T cells and B cells	Activated T and B cells	Inhibit T and B cell proliferation, inhibit haematopoiesis, promote wound healing

Abbreviations: BM, bone marrow; DCs, dendritic cells; G-CSF, granulocyte-colony stimulating factors; M-CSF, macrophage colony stimulating factor; Th, T helper cells.

**Table 3:** Compilation of various electrochemical-based detection techniques used and respective criteria along with results.

Technique	Sample	Volume	Substrate	Analyte	Limit of detection	Range of detection	Reference
Voltammetry	Serum	20 µL	Whatman No.1 chromatography paper	CEA	2 pg mL^−1^	0.01-500 ng mL^−1^	[85]
	Serum	20 µL	Whatman No.1 chromatography paper	NSE	10 pg mL^−1^	0.05-500 ng mL^−1^	[85]
Impedance Spectroscopy	Serum	25 µL	Whatman No.1 chromatography paper	IFN-γ	3.4 pg mL^−1^	5-1000 pg mL^−1^	[86]
	Serum	N.A.	Whatman No.1 chromatography paper	CEA	2.68 ng mL^−1^	6-20 ng mL^−1^	[36]
Electrochemiluminescence Detection	Aptamer bioconjugates	20 µL	Whatman No.1 chromatography paper	HL-60 cells	56 cells per mL	56 to 5.6 × 10^6^ cells per mL	[90]

Abbreviations: N.A., not available.

**Table 4:** Advantages and disadvantages of paper-based biosensors over ELISA-based sensing platforms.

**Property**	**Advantage**	**Utility**
Sensitivity	High	Competitive with modern instrumental methods
Specificity	High	Competitive with modern instrumental methods
Reproducibility	High	Competitive with modern instrumental methods
Detection Limit	Low	Competitive with modern instrumental methods
Disposable	Yes	Convenience in handling
Response Time	Fast	Able to obtain results within seconds or minutes
Cost	Low	Able to use in developing countries with limited resources as point-of-care diagnostic devices
**Property**	**Disadvantage**	**Circumvention**
Reusability	No	-
Stability of stored biomolecule	Few weeks if not protected	Fabricated sensor must be stored in dry state and in sealed polybags
